# Editorial: The diagnosis and the therapy of social cognition deficits in adults affected by ADHD and MCI

**DOI:** 10.3389/fneur.2023.1162510

**Published:** 2023-03-02

**Authors:** Leonardo Sacco, Lucia Morellini, Chiara Cerami

**Affiliations:** ^1^Neuropsychological and Speech Therapy Unit, Neurocenter of Southern Switzerland, Ente Ospedaliero Cantonale (EOC), Lugano, Switzerland; ^2^Faculty of Biomedical Sciences, Universitá della Svizzera italiana, Lugano, Switzerland; ^3^IUSS Cognitive Neuroscience (ICoN) Center, University School for Advanced Studies IUSS-Pavia, Pavia, Italy; ^4^Cognitive Computational Neuroscience Research Unit, Mondino Foundation IRCCS, Pavia, Italy

**Keywords:** MCI, ADHD, empathy, social cognition (SC), Alzheimer's disease

Social cognition is a multidimensional construct (1, 2) where different cognitive components converge with the aim of efficiently recognizing and interpreting information acquired from the social environment and of producing effective social interactions. Social interactions can be explained through three distinct processes: (1) *social perception*, the ability to distinguish facial expressions, voice or gesture information (3); (2) *social understanding*, including empathy and theory of mind (ToM) skills, namely the ability to share (affective empathy) and infer (cognitive empathy) emotions in self and others (4) and the ability to represent mental states and feelings of self and others (1); (3) *social decision-making*, the ability to adapt social behavior to social context (5). Social cognition is thus a fundamental process for humans in daily life. Accordingly, the DSM-5 (6) included socio-cognitive skills among the 6 main neurocognitive domains of functioning. This complex cognitive domain can be affected in both neurodevelopmental, e.g. Attention-deficit/hyperactivity disorder (ADHD) (7), and neurodegenerative disorders, e.g. Mild Cognitive Impairment (MCI), Alzheimer's dementia (AD) and frontotemporal dementia (FTD) (8–10). Especially for ADHD patients, social cognition deficit can contribute in daily living impairments, particularly in work, health and academic domains. Patients affected by ADHD are also at high risk of developing personality disorders (11). In light of what was mentioned before, it is necessary an intervention to empower social cognition skills in order to improve the quality of life on individuals with ADHD.

## Diagnosis of social cognition deficits in adults affected by ADHD and MCI

A large body of evidence consistently supports an impairment of social cognition not only in children and adolescents with neurodevelopmental disorders [see (12), for a review] but also in adults with ADHD (Morellini et al.). Literature shows however inconclusive and ambiguous results mainly due to methodological inaccuracy and variability, as small sample size, and lack of standardized and harmonized protocols in acquisition and collection of data (Morellini et al.). Findings in MCI and dementia proved a variable degree of impairments in affective and cognitive facets of social cognition domain [see (8, 13)]. MCI performances significantly vary among studies according to inclusion criteria and global cognitive profile (13) proving even opposite socio-cognitive performances [e.g. (14, 15)]. Socio-cognitive disorders have been reported in MCI patients compared with healthy control subjects (10, 16–18), using emotion recognition test [i.e., Ekman 60 faces test (19)], and affective and cognitive ToM tasks [i.e., Reading the Mind in the Eye (20); The Awareness of Social Inference Test (21); the Story-based Empathy Task (2); the Social Cognition and emotional assessment (22)]. Crucially, longitudinal trajectories of changes in social cognition abilities are still largely unknown in MCI patients. The 12-months longitudinal study of Rossetto et al., that is part of this Research Topic, is pioneering in this field. As shown by the authors, affective ToM performances worsen earlier than cognitive ToM ones. As measured by the Reading the Mind in the Eye (20) and the Strange Stories test (23), at 12 months the 46% of MCI was impaired in affective ToM compared to the 28% in cognitive ToM (Rossetto et al.).

In view of what mentioned above, there is a strong need of better experimental studies and better clinical neuropsychological tools for the evaluation of social cognition performances in neurodevelopmental and neurodegenerative patients. This is a crucial issue in clinics, especially for an accurate early detection of subtle changes in social behavior as those that may occur in MCI or in the prodromal phases of FTD (9, 10). Nonetheless, it has also main implications in research since better experimental tasks may easier capture novel socio-cognitive profiles and longitudinal trajectories of damage. Recently, a European harmonization initiative (24) recommended a Uniform DataSet for the neuropsychological assessment of MCI patients. This includes a social cognition test (i.e., the Story-Based Empathy Task) in order to cover each main cognitive domain in the standard neuropsychological battery, as suggested by DSM-5. Nonetheless, there is a huge debate in the literature on which instrument or combination of tasks may reliably detect socio-cognitive deficits in neurocognitive patients (25, 26). Though the large amount of research evidence collected in the last two decades, only a limited number of neuropsychological instruments (e.g., Ekman 60 faces test, Reading the Mind in the Eye, Social Cognition and Emotional Assessment), in addition to the Story-based Empathy Task have been validated for clinical use. In this Research Topic, a validation of another social cognition instrument (i.e., the 48-Item Yoni short version) has been made in order to investigate ToM in the Italian-speaking population (Isernia et al.).

## Treatment of social cognition deficits in adults affected by ADHD and MCI

Socio-emotional impairments may have significant effect on the patient-caregiver dyad (27–29). Better awareness of emotion recognition and processing deficits in neurocognitive patients has been proved to mitigate caregiver burden distress and improve patient homecare setting (30). Although this evidence, treatments specifically targeting socio-emotional alterations in NCDs are crucially lacking and studies are of medium-low quality (31). Oxytocin is the only drug specifically tested with promising results in improving social behaviors and cooperative behavior in frontotemporal dementia (31). Long-term efficacy as well as effects in other neurocognitive disorders have not been fully elucidated. More recent advances in treatment strategies in MCI patients focus on other pharmacological and non-pharmacological therapeutic interventions. Anti-amyloid antibodies as Aducanumab, an amyloid beta-direct monoclonal antibody that has been approved in the US for early stages of AD (32), or Lecanemab, a humanized monoclonal antibody that promotes clearance of protofibrils implicated in AD pathogenesis (33), have focused general attention in the last years. Unfortunately, there is still poor high-quality evidence supporting an effective pharmacological treatment for MCI (34) and additional clinical trials are certainly needed to further investigate the efficacy and safety of these treatments (34). In view of the need of limiting the increasing risk of conversion to dementia (35), combined strategies like physical exercise and cognitive training have also been applied with good efficacy improving executive functions and global functioning in patients with MCI (36). Among the emerging therapeutic strategies for MCI, non-invasive brain stimulation techniques as Trans-cranial Magnetic Stimulation (TMS) and trans-cranial Direct Current Stimulation (tDCS) are gaining increasing interest. TMS would appear to be better tolerated and more effective than tDCS in improving global cognitive functioning in MCI (37). Other non-pharmacological therapeutic strategies have been tested, as acupuncture treatments, well summarized by the meta-analysis drawn up by Zihan et al. and included in this Research Topic (Yin et al.). Regarding ADHD therapy there are meta-analysis, including more than 190 clinical randomized trials, supporting the efficacy of pharmacological and non pharmacological therapy (38) as well as a Cochrane review (39). However, there is a lack of data in adults and the effect of such therapy in the domain of social cognition remains unspecified.

In conclusion, ADHD and MCI are disorders that need better diagnostic tools and new treatment options. There is however a long way to go to identify effective neuropsychological tools in clinics and to test therapeutic targets to treat social cognition changes in such disorders for the ultimate benefit of patients and caregivers.

## Author contributions

LS and LM wrote the manuscript. CC revised the manuscript. All authors approved the submitted version.
